# Laparoscopic omental flap for the treatment of thoracic aortic graft infection: report of two cases and review of the literature

**DOI:** 10.1186/s13019-020-01146-7

**Published:** 2020-05-29

**Authors:** Jiawei Zhou, Junfeng Sun, Xingxing Yao, Guochang Zhao, Fuqiang Sun, Weiyong Sheng, Fanfan Lu, Haibo Zhan, Chao Liu

**Affiliations:** 1 Department of Cardiovascular Surgery, First Affiliated Hospital of Zhengzhou University, Zhengzhou, China; 2 Department of Gastrointestinal Surgery, First Affiliated Hospital of Zhengzhou University, Zhengzhou, China

**Keywords:** Aortic graft infection, Laparoscope, Omental transposition

## Abstract

**Background:**

Thoracic aortic graft infection (TAGI) is a rare and serious complication after surgery for which the treatment is controversial and challenging. Rather than following the traditional surgical strategy of graft replacement and extensive debridement, we have chosen to preserve the graft and cover it by a laparoscopic omental flap. In this article, we describe the clinical manifestation, diagnostic modalities, and treatment of this disease and analyze the role of laparoscopic omental flaps in its treatment.

**Case presentation:**

We present two cases of thoracic aortic graft infections that had undergone surgical graft replacement for acute Stanford type A dissection. Their clinical manifestation of infection was atypical, with computed tomography suggesting infection of the grafts. Both patients were successfully treated with debridement, laparoscopic omental transposition, and antibiotics. The first case, a 55-year-old male, was found to have an infection at the aortic arch. The second case is a 52-year-old male who was found to have infection at the ascending aorta and arch. Surprisingly, both intraoperative cultures were negative. The infections were brought under control and the patients recovered steadily after surgery. Early follow-up results showed no signs of graft infection.

**Conclusion:**

These findings suggest that graft replacement for the treatment of TAGI is not always necessary in selected patients. Conservative surgical treatment, including laparoscopic omental transposition, is effective and less invasive for treating TAGI.

## Background

Thoracic aortic graft infection **(**TAGI) is a rare but severe complication after cardiac surgery with thoracotomy. Graft infections are associated with aortic valve insufficiency, peripheral arterial embolus, false aneurysm, rupture, chronic draining of external sinuses, aortocutaneous fistula, aortobronchial fistula, hemolysis, graft deformity, chronic sepsis, septicemia, shock, and death [1]. Most complications are fatal. Graft infection has a high mortality rate and is extremely challenging for patients and surgeons. TAGI is complicated and risky to treat because of its anatomical location and adjacent vital organs. For now, treatments for TAGI are controversial. Traditionally, radical debridement and repeated in situ graft replacement has been used for management. However, these are risky and not always necessary [2]. Here, we report a conservative method in which we applied laparoscopic omental flaps to treat two patients with TAGI after type A aortic dissections, which reduced the surgical risk and achieved favorable results in early follow-up. Few similar procedures throughout the world have been reported.

## Case presentation

### Case 1

A 55-year-old man was readmitted to our institute for poor chest wound healing on May 25, 2019. Four weeks previously, he had undergone a Bentall procedure using a No. 23 aortic valved graft (St. Jude Medical Inc, MN, USA) and a replacement of the ascending aorta and total arch with a 28-mm Dacron graft (Terumo Corporation, Tokyo, Japan) and implantation of a frozen elephant trunk (Cronus, Microport, Shanghai, China) under deep hypothermic circulatory arrest and selective antegrade cerebral perfusion for acute Stanford type A dissection. We used two chest plates to the fixed sternum in a closed procedure. He was finally discharged with a healed wound. Physical examination upon readmission showed a sternal upper middle section of skin ulcer about 2 × 4 cm, the chest exposed with a yellow purulent secretion, and normal temperature (36.8 °C). Laboratory investigation revealed mild leukocytosis of 10.8 × 10^9^ cells/L.

Healing was slow despite supportive therapy and debridement. The ulceration was found to be concentrated on the upper part of the sternum plate. Considering that the patient’s failure of wound healing was caused by an allergic reaction to the sternum plate, the sternum plate was taken out, the necrotic tissue was cut off, and the wound healed completely. However, a 1-cm fluctuant area developed in the upper part of the healed sternotomy incision. A few days later, a copious amount of yellow purulent fluid was drained and formed a sinus tract. Specimens from the pus collection cultures for bacterial screening were negative.

**Fig. 1:**
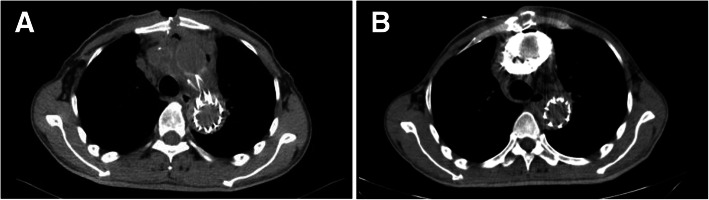
**a** Computed tomography (CT) shows soft tissue and ectopic gas surrounding the ascending aortic graft having a diameter of 14 mm. **b** CT scan showing a large amount of contrast agent wrapped around the replacement ascending aorta graft with a small amount of gas

We then performed a sinogram. The contrast agent was injected into the sinus tract until overflow. Then a 64-slice chest computed tomography (CT) scan was performed to determine the direction, length, and range of the sinus tract. CT examination showed that no graft was involved. Then we continued to plug the drainage strip after debridement every day. The sinus tract was still challenging to heal. CT showed soft tissue and ectopic gas surrounding the ascending aortic graft having a diameter of 14 mm at 7 weeks after surgery (Fig. 1, a). A repeated sinogram showed a large amount of contrast agent wrapped around the replacement ascending aorta graft with a small amount of gas (Fig. 1, b). The image raised the possibility of aortic graft infection. Considering the severe consequences of graft infection and the patient’s condition, we performed thorough debridement and emergency laparoscopic greater omental transplantation combined with gastrointestinal surgery. Intraoperative findings were that the substernal adhesion was serious, and some white necrotic material could be seen around the ascending aorta graft.

We harvested the omental flap by laparoscopic instruments. The pneumoperitoneum was then treated and we maintained pneumoperitoneum pressure. A telescope and three 5-mm Trocars were placed properly. The greater omentum was detached from the transverse colon by ultrasound scissors, and a bulky omental flap was created. The infected and necrotic tissues, especially around the aortic graft, were thoroughly removed, and intraoperative tissue specimens were submitted to bacterial culture. The mediastinum was repeatedly washed with normal saline and povidone-iodine solution, and iodine solution was soaked around the graft. A 5-cm substernal diaphragm was incised, and the greater omentum was pulled to the mediastinum through the diaphragm with forceps. Great care was used in making the greater omentum pedicle in the abdominal cavity in a state of no tension.

The greater omentum was segmented and wrapped around the aortic graft to fill dead space. The sternum was fixed with a steel wire, the upper end of which was indwelled with a posterior sternal rinse tube. Two chest tubes were inserted under the xiphoid process and around the graft. After the operation, gentamycin rinse was continuously provided with saline and the patient was treated with intravenous antibiotics. Bacterial cultures were negative. The wound healed well after the surgery, and the patient recovered steadily. The patient was discharged 2 weeks after the reoperation. As of follow-up at 3 months, the patient is doing well. CT examination of the chest has indicated no signs of infection in the area of the aortic prosthesis or elsewhere in the chest.

### Case 2

A 52-year-old man was admitted to the hospital due to skin ulceration of the upper sternum on July 22, 2019. Three years previously, the patient had undergone the same procedure as in case 1 for type A aortic dissection in our hospital. The difference was the use of a 25-mm composite graft (Medtronic Inc, Minneapolis, MN, USA) in the Bentall procedure. Physical examination on readmission showed a skin ulcer on the upper sternum of about 1 × 1 cm with pale yellow purulent discharge. The patient’s body temperature was normal (37.1 °C), and the white blood cell count was 8.1 × 10^9^ cells/L.

**Fig. 2:**
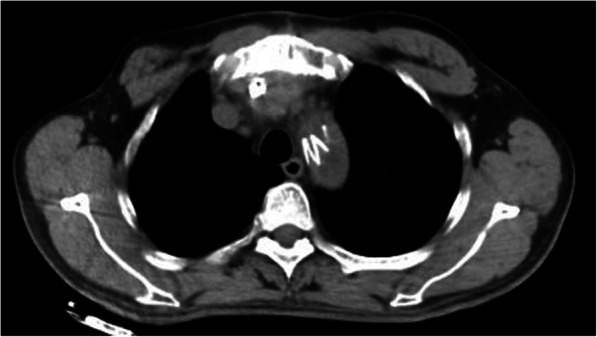
CT scan showing a little contrast agent surrounding the displaced aortal arch with a bit of gas

**Fig. 3:**
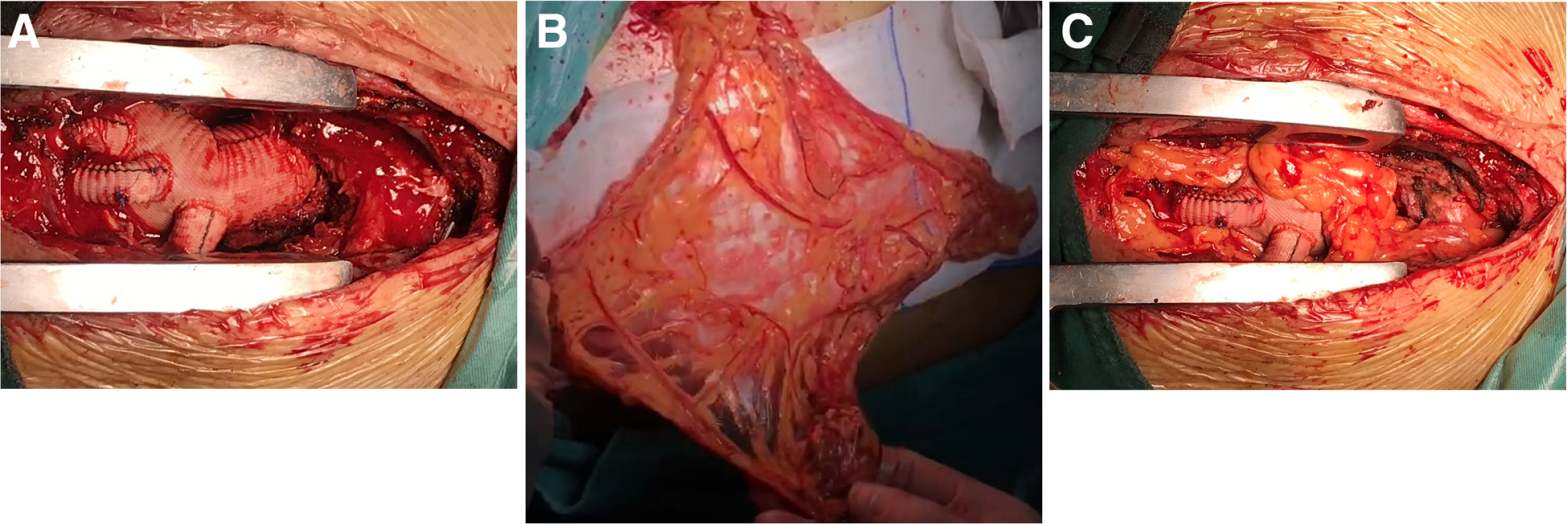
Intraoperative photograph of case 2. **a** Infected aortic graft surround by black peptone-like plasma and necrotic tissue. **b** A pedicle of the omentum is bulky and well vascularized. **c** The omental flap fills the mediastinal cavity and wraps around the infected aortic graft

The patient was treated with debridement daily. However, there was no progress in wound healing. The skin of the upper sternum formed a sinus tract, and the first sinogram with CT showed that infection did not involve the aortic graft. We continued to plug a drainage strip with daily dressing change, but the tract was slow to heal. Bacterial cultures were negative. The second sinogram showed some contrast agent surrounding the displaced aortal arch with a bit of gas (Fig. 2). Thorough debridement and laparoscopic greater omental transplantation were also performed (Fig. 3). Intraoperatively, large amounts of black peptone-like plasma and necrotic tissue were found around the ascending aorta and aortic arch. Intraoperative specimens were submitted for culture. Postoperatively, continuous antibiotics with saline rinse and intravenous antibiotic treatment were provided. The intraoperative specimen bacterial cultures were also negative. The wound healed well after the operation, and the patient recovered gradually. As of follow-up at 2 months, the patient is doing well. CT examination of the chest has indicated no signs of infection in the area of the aortic prosthesis or elsewhere.

## Discussion

TAGI after surgery is a rare but serious complication, with an incidence of approximately 1–3% [3] and mortality of 25–75% [4]. Patients with aortic surgery have an increased chance of postoperative infection due to reduced immune defenses [1]. Additionally, the poor anti-infection ability of grafts further increases the chance of postoperative infection. Postoperative infection seriously affects the benefits of surgery, bringing significant psychological and economic burden to patients and sometimes having disastrous consequences. Once diagnosed as “graft infection,” emergency surgery is required to avoid fatal complications.

The primary causative pathogens are gram-positive organisms such as *Staphylococcus aureus* [5]. Pineda et al. [6] reported two cases of TAGI that were secondary due solely to *Propionibacterium* species. The most common etiology of graft infection is usually a surgical site infection (SSI), followed by seeding of distant infection sites [7]. The two patients in the present report likely had infection secondary to the SSI. Therefore, such infections are likely of great importance.

The diagnosis of TAGI requires a multidisciplinary approach. Contrast-computed tomography (CT) is effective and can show perigraft fluid at low density, ectopic gas, perigraft structures with an increased amount of soft tissue (> 5 mm) between the graft and the surrounding sac, and pseudoaneurysm formation [8]. CT-guided aspiration of cavity perigraft fluid collection plays an important role in differentiating abscess formation from seroma or hematoma. Some reports indicate the usefulness of fused fluorodeoxyglucose-positron emission tomography (FDG-PET)/CT to identify the site of prosthetic vascular graft infection [9]. Besides clinical symptoms such as fever, sweats, and chills, biomarkers play a critical role in auxiliary diagnosis, including C-reactive protein (CRP) and elevated white blood cell counts. The diagnosis of TAGI is supported mainly by bacterial culture. However, tissue or blood cultures obtained from such patients are negative in a third of them [4]. Timely diagnosis is essential for proper treatment.

There are various treatments for TAGI that are mainly divided into repeated in situ graft replacement and graft-sparing strategies. Historically, patients have been treated with radical debridement and in situ graft replacement. However, graft replacement is not available or practical in routine clinical practice, and it is also associated with severe complications and high mortality. In 1984, Hargrove et al. [10] reported successful infection control by omental filling without the removal of infected prosthetic grafts. This approach has produced favorable results. Nakajima et al. [11] have modified this approach in two steps and reported a 100% survival rate and a 100% recurrence prevention rate in five patients.

The conservative approach of thorough debridement and greater omentum wrapping for revascularization is more practical. The key to the operation is the removal of infected and necrotic tissues and local revascularization. If the local blood supply is not enough, the infection will be difficult or worse to control, especially in patients with grafts. After debridement, the omentum, pectoralis major muscle flap, or rectus muscle flap can be used to fill the retrosternal defect. We used the omentum in both present cases. The omentum is a bulky, well-vascularized flap that can reach the base of the neck and fill the sternal defect. It has a strong anti-infection and repair function and can treat the defect well. Because of its special physiological functions, the greater omentum has been widely used in thoracic surgery, breast surgery, for burns, and in other fields [12], and its unique advantages have been proven in long-term clinical practice. It also shows good results in cardiac surgery [13]. The flexibility of the omentum is particularly suitable for filling irregular spaces around the graft to eliminate the dead space as thoroughly as possible.

With the development and popularization of laparoscopic technology, we creatively adopted laparoscopy to obtain a greater omentum pedicle, which has the advantages of minimally invasive incision, favorable appearance, and fast postoperative recovery compared with traditional laparotomy [14]. We found that the drainage volume was less than the flushing volume after the continuous application of antibiotic irrigation, which also indicated the strong absorptive capacity of the greater omentum. Antibiotic therapy has a great impact on the prognosis. Specific antibiotics are selected by drug sensitivity testing and clinical experience.

Although the bacterial culture results of case 1 and case 2 were negative, intraoperative findings combined with preoperative CT images still indicated a diagnosis of TAGI. Early literature indicated that a considerable number of patients (> 90%) show positive intraoperative microbiological specimens [4]. However, negative reports for intraoperatively obtained microbiological specimens do not exclude graft infection. Case 1 and case 2 were early and late graft infections, respectively, with atypical clinical manifestations.

Laparoscopic greater omental transplantation for the treatment of TAGI has been rarely reported around the world, though it has the following advantages: (1) There is relatively little surgical trauma, it is simple and easy to perform, and it is easily accepted by most elderly and frail patients. (2) The omentum is rich in vascular and lymphatic tissue, which gives it a strong ability to resist infection and repair. Auxiliary washing and systemic antibiotic treatment can maximize the control of infection. (3) The greater omentum has more bulk and stronger compliance than muscle flaps, and it can fill defects fully. (4) A laparoscopic omental flap reduces hemorrhage and the incidence of postoperative abdominal complications. However, disadvantages include (1) the abdominal cavity loses the function of the omentum to envelop inflammation, leading to peritonitis in the case of acute abdominal diseases, and (2) the infected area communicates with the abdominal cavity through the omentum, and there is a risk of abdominal infection. Neither patient in the present study had an abdominal infection.

Some of our recommendations include the following: (1) Stop warfarin in time, and intravenous vitamin K antagonism is necessary to avoid excessive hemorrhage. (2) After anesthesia, diluted methylene blue can be injected through the sinus orifice for sinus tract staining to define the scope of the sinus tract and facilitate intraoperative debridement. (3) The sternum should be split as completely as possible during the operation. (4) Great care should used during debridement, because the graft becomes brittle after infection.

## Conclusion

TAGI presents a serious challenge to surgeons, and its management is still controversial. The greater omentum plays an important role in conservative treatment. Conservative surgical treatment, including laparoscopic omental transposition, is effective and less invasive for treating TAGI.

## Data Availability

Not applicable.

## Abbreviations

TAGI: Thoracic aortic graft infections
CT: Computed tomography
SSI: Surgical site infection
